# Islands Promote Diversification of the Silvereye Species Complex: A Phylogenomic Analysis of a Great Speciator

**DOI:** 10.1111/mec.17830

**Published:** 2025-06-11

**Authors:** Andrea Estandía, Nilo Merino Recalde, Ashley T. Sendell‐Price, Dominique A. Potvin, William Goulding, Bruce C. Robertson, Sonya Clegg

**Affiliations:** ^1^ Department of Biology, Edward Grey Institute of Field Ornithology University of Oxford Oxford UK; ^2^ School of Science, Technology and Engineering University of the Sunshine Coast Petrie Queensland Australia; ^3^ School of Environment and Science Griffith University Nathan Queensland Australia; ^4^ Biodiversity Program Queensland Museum Kurilpa Brisbane Queensland Australia; ^5^ Department of Zoology Otago University Dunedin New Zealand

**Keywords:** dispersal, islands, passerine, phylogeny, radiation, speciation, *Zosterops*

## Abstract

Geographic isolation plays a pivotal role in speciation by restricting gene flow between populations through distance or physical barriers. However, the speciation process is complex, influenced by the interplay between dispersal ability and geographic isolation, as seen in “great speciators” – bird species that simultaneously have broad island distributions but high levels of subspecific diversity. Comparing genomic population differentiation in species that occupy both continental and island settings can reveal the effects of different forms of geographic isolation and validate if the primary mechanism proposed to catalyse a great speciator pattern, that is, dispersal reduction following island colonisation, has occurred. The highly diverse white‐eye family Zosteropidae includes several great speciators, including the silvereye ( *Zosterops lateralis* ), with 16 subspecies (11 occurring on islands), distributed on the Australian continent and numerous southwest Pacific islands. We compared continental and island patterns of divergence using whole genome and morphological data. Australian mainland populations showed a low genetic population structure, lack of isolation by distance patterns and low morphological diagnosability, suggesting that the species' dispersal propensity in a continental setting is sufficient to overcome multiple forms of geographic barriers and large geographic distances. In contrast, except for island populations less than 200 years old, most island populations were highly genomically structured with clearer morphological diagnosability even if separated by relatively short geographic distances. The inferred reduction of dispersal propensity in island situations is consistent with the proposed model of great speciator formation on islands. Our phylogenomic analyses also allowed resolution of the silvereyes' evolutionary position, showing their relatively early emergence (~1.5 Mya) within the rapidly radiating Zosteropidae, while population‐level analyses demonstrated where morphological subspecies and genomic data align and disagree. However, the silvereye example also shows how uncertainties about relationships remain when reconstructing evolutionary history in rapidly radiating groups, even when whole genome data is available. Altogether, our results show how within‐species genomic and morphological patterns measured over broad spatial scales and with varying geographic contexts can help reveal when particular stages of speciation such as great speciators are likely to emerge.

## 1 Introduction

Geography plays a key role in the speciation process by limiting allele exchange across distant and isolated populations, resulting in divergence (Anderson and Weir 2022; Bolnick and Fitzpatrick 2007; Coyne and Allen Orr 1998). Isolation‐by‐distance patterns, whereby individuals that are geographically close to each other are likely to be more related than those farther apart, create a gradient in population genetic structure (Wright 1943), while geographical barriers, such as mountain ranges, deserts, or water gaps, can align with more abrupt divergence patterns (White 2016). The phenotypic and genomic divergence patterns that emerge are contingent on a species' dispersal capacity (Kisel and Barraclough 2010), a trait that can itself change in different geographic situations (Diamond 1981; Komdeur et al. 2004). On the one hand, highly dispersive species with broad distributions might be expected to maintain population connectivity and gene flow even across large distances and barriers (Andersen et al. 2015; Bohonak 1999; Suárez et al. 2022). Yet, even for terrestrial species with perceived high dispersal capacity such as flighted birds and bats, water gaps can be particularly effective at limiting gene flow (Emerson 2002), sometimes over relatively short geographic distances (Cowles and Uy 2019; Diamond 1977). In the absence of direct measures of dispersal, the population genetic structure can act as a proxy for connectivity and hence dispersal. Examination of population genetic structure of a single taxon across multiple geographic situations is therefore particularly useful to understand how geographic distance and barriers of different types affect dispersal and how their interplay shapes divergence patterns.

One evolutionary divergence pattern where information from multiple geographic contexts would be particularly informative is that of the ‘great speciators’. As applied to island avifauna, the original term described bird species in Northern Melanesia that inhabit multiple islands and exhibit numerous subspecies (Diamond et al. 1976; Mayr and Diamond 2001). Mayr and Diamond (2001) proposed specific criteria for identifying such species, including having more than seven subspecies or between four and six subspecies distributed across several islands. More recent work recognises that this approach is rather limited and overly simplistic and has suggested that great speciators should be viewed as part of a continuum rather than a discrete category (Estandía 2023). The formation of a great speciator pattern is thought to involve two key phases: an initial expansion facilitated by high dispersal propensity across multiple islands, followed by reduction in dispersal, population differentiation and (sub)speciation (Diamond 1981; Dufour et al. 2024). These phases also align with the early stages of the taxon cycle, a process in which island species undergo cyclical phases of range expansion and contraction (Wilson 1961). However, theoretical modelling and empirical data show that species richness and speciation rates are highest when dispersal is intermediate—not so high as to result in substantial gene flow and not so low as to limit opportunities to occupy new niches (Ashby et al. 2020; Claramunt et al. 2011; Yamaguchi 2022). Following from this, intermediate dispersal within a species could maximise the number of subspecies and the range of their island distribution, creating a great speciator pattern without needing to invoke a change from high to low dispersal. Because the great speciator concept was derived from island‐dwelling species and studies since have focused almost exclusively on island forms (DeRaad et al. 2023; Jønsson et al. 2014; Manthey et al. 2020), it is difficult to say if dispersal propensity of an island‐distributed taxon is different to that expected in other geographic contexts. We can examine this when an insular great speciator is also represented by continental populations.

A fundamental step in comparing divergence patterns between island and continental settings and to understand when great speciator patterns are likely to arise is to have a robust understanding of evolutionary relationships among forms. Traditionally, phenotypic traits were used to reconstruct evolutionary relationships, but genetic studies have revealed that morphological species and subspecies frequently do not align with phylogenetic relationships (Hirano et al. 2014; Keating et al. 2023). However, using relatively short segments of DNA to reconstruct phylogenies may also fail to resolve evolutionary relationships. Genomic approaches now allow us to reconstruct evolutionary relationships with increased confidence (Lee and Palci 2015), although it is still challenging for those clades that radiate rapidly, such as many great speciators (Moyle et al. 2009). The condensed sequence of cladogenetic events and increased levels of incomplete lineage sorting (ILS) driven by rapid radiation (DeRaad et al. 2023) and recent divergence (Irestedt et al. 2013) complicate phylogenetic inference (Meleshko et al. 2021). Additionally, gene flow can lead to reticulate evolution (Xu 2000). Additionally, a deep and comparable taxon sampling is needed to disentangle evolutionary histories, especially when they are complex histories (Stervander et al. 2015). Therefore, it is important to use a combination of multiple phylogenetic approaches to identify conflicting nodes (Smith et al. 2020) and dense sampling across the genome to provide reliable phylogenetic reconstructions where sources of error are appropriately handled (Kapli et al. 2020).

One species with a wide continental and insular distribution in the southwest Pacific is the silvereye ( *Zosterops lateralis* ). This species belongs to the white‐eye family, Zosteropidae, which originated in southeast Asia (Gwee et al. 2020), and rapidly expanded into the Palaeotropics and Oceania within the last two million years (Moyle et al. 2009), giving rise to over 120 species (Clements et al. 2021). The silvereye has at least 16 morphological subspecies, 11 on islands and the remainder on the Australian continent (Mees 1969), and its broad distribution and diversity in the form of morphological subspecies fit the great speciator concept. The divergence of silvereye subspecies on islands was assumed to represent multiple independent colonisations originating from the Australian mainland (Mees 1969; Black 2010). Phenotypic divergence of island‐dwelling silvereyes occurs rapidly, and water barriers appear to be important in the arrangement of morphological subspecies (Clegg et al. 2008; Estandía, Sendell‐Price, Oatley, et al. 2023; Radu et al. 2024; Sendell‐Price et al. 2021). On the Australian mainland and Tasmania, five morphological subspecies of silvereye are recognised (Mees 1969; Schodde and Mason 1999). Banding records show that silvereyes can disperse long distances (Figure S1) and two subspecies (*Z. l. lateralis* and *Z. l. westernensis*) display partial migration (Chan 1994)). There is evidence of substantial gene flow among eastern Australian subspecies (Estandía, Sendell‐Price, Oatley, et al. 2023; Potvin et al. 2013), but relationships with other mainland subspecies are unclear. There are multiple potential biogeographic barriers that affect divergence and subspecies patterns in continental Australian passerines (Dolman and Joseph 2015). However, the silvereyes' geographically wide distribution and subspecific variation in dispersal tendency might reduce the impact of continental biogeographic barriers seen in other passerines. As a result, we might expect less pronounced genetic and phenotypic differentiation among continental subspecies compared to their island counterparts.

We sequenced the whole genomes of 114 individuals from 12 silvereye subspecies from islands and the Australian continent and from six other white‐eye species. We investigated the phylogenomic relationships of silvereyes with three main objectives: (i) to reconstruct their evolutionary history; that then provides the basis to (ii) compare connectivity levels between island and continental populations, which allows us to test whether islands reduce gene flow more than equivalent continental distances or geographical barriers; and (iii) to test whether different geographic contexts (island vs. continental settings) drive phenotypic and genetic population differentiation, particularly in relation to the emergence of the great speciator pattern. This approach allowed us to explicitly test whether island populations originated through independent colonisations directly from the mainland or via successive colonisations between islands.

## 2 Methods

### 2.1 Field Sampling

**FIGURE 1:**
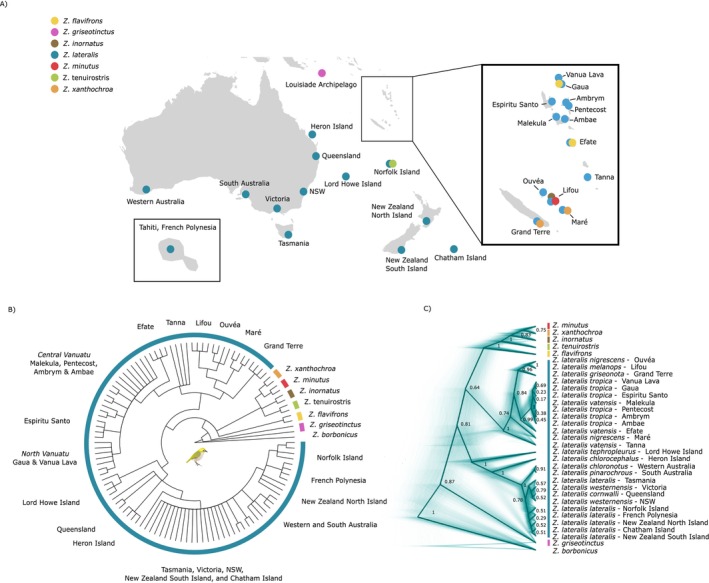
(A) Geographic distribution of sampled individuals sequenced in this study, with locations marked and coloured by species. Each point represents a sampling site, with approximately four individuals per location, though exact numbers vary (see Table S1 for details). The inset highlights sampled locations in French Polynesia. White asterisks represent the recently colonised populations. (B) Phylogenetic relationships across South Pacific white‐eyes, including a dense sampling of silvereye subspecies using a coalescent‐based approach with SVDquartets. (C) A DensiTree representing the variability in the inferred phylogenetic relationships inferred with SNAPPER, highlighting areas where the tree space is more densely populated. The root canal, or the phylogeny with the highest clade support, is highlighted by the darkest lines.

We sampled seven South Pacific white‐eye species (the silvereye *Z. lateralis*, representing 12 subspecies, the Vanuatu white‐eye *Z. flavifrons*, the Louisiade white‐eye *Z. griseotinctus*, the large Lifou white‐eye *Z. inornatus*, the small Lifou white‐eye *Z. minutus*, the slender‐billed white‐eye *Z. tenuirostris* and the green‐backed white‐eye *Z. xanthochroa* ) (Table S1; Figure 1A) and included a published Réunion grey white‐eye *Z. borbonicus* sequence (Leroy et al. 2021) which served as an outgroup. Birds were caught in the wild using mist nets or hand‐operated traps and released at the site of capture following processing. A blood sample of 20–40 μL was collected from the brachial wing vein and stored in 1 mL of Queen's lysis buffer (Seutin et al. 1991) or 100% molecular grade ethanol. We recorded a suite of morphological measurements: wing length (maximum flattened chord of the longest primary feather measured with a metal ruler), tail length (the length of the central tail feathers from the base to the tip measured with dividers), metatarsal length, head length from the rear of the skull to the bill tip, and bill length, width and depth at the posterior nostril opening, all measured with dial callipers.

### 2.2 DNA Extraction, Library Preparation and Sequencing

We extracted DNA from blood samples using a standard phenol‐chloroform protocol (Seutin et al. 1991). We added 20 μL of blood sample to a microcentrifuge tube containing 250 μL of DIGSOL extraction buffer (0.02 M EDTA, 0.05 M Tris–HCl (pH 8.0), 0.4 M NaCl, 0.5% sodium dodecyl sulphate (SDS)) and 10 μL of Proteinase K (20 mg/mL). Samples were incubated at 55°C overnight. Following incubation, we added 250 μL of phenol:chloroform:isoamyl alcohol (25:24:1), gently mixed the samples for 10 min and then centrifuged at 10,000 rpm for 10 min. We transferred the aqueous layer to a new microcentrifuge tube and repeated the previous step by adding the phenol mixture, mixing, and centrifuging. We recovered the aqueous phase, transferred it to a new tube, added 250 μL of chloroform:isoamyl alcohol (24:1) and mixed and centrifuged the samples as in the previous steps. We precipitated the DNA by adding 2 volumes of ice‐cold 100% ethanol, 1 volume of 2.5 M ammonium acetate and 2 μL of glycogen, leaving it overnight at −20°C, and then centrifuging the tubes for 10 min at 15,000 rpm at 4°C before removing the supernatant. We rinsed the precipitate with 500 μL of ice‐cold 70% ethanol and centrifuged the tubes as in the previous step. We removed the supernatant and left the precipitated DNA to dry at room temperature. Once dried, the DNA was resuspended in 50 μL of TE (Tris‐EDTA) buffer. We quantified the DNA concentration with a Qubit 2.0. DNA and extracts were sent to Novogene UK for library preparation and whole genome sequencing at ~5X on the Illumina Novaseq 6000 platform (Illumina, San Diego) using paired‐end 150 bp sequencing reads.

### 2.3 Quality Control and Genotype Calling

We trimmed reads to remove adapter content and base calls of low quality using *fastp* (Chen et al. 2018) with default settings and 10 bp trimmed from the start of each read. We aligned cleaned reads of each individual to a pseudo‐chromosome assembly for this species (Estandía, Sendell‐Price, Oatley, et al. 2023) using the Burrows‐Wheeler Aligner (BWA)‐mem algorithm (Li and Durbin 2010). We calculated genotype probabilities in ANGSD (Korneliussen et al. 2014) using the samtools model and keeping SNPs with a minimum allele frequency of 0.05 and a mapping quality score ≥ 20. We also removed any loci putatively under selection detected with PCAngsd (Meisner and Albrechtsen 2018) to have an unbiased neutral dataset that allows accurate population structure analyses (Kirk and Freeland 2011). We further filtered our dataset by only retaining those sites situated on autosomes, excluding a neo‐sex chromosome resulting from a translocation of the ancestral chromosome 4A and the W and Z chromosomes (Leroy et al. 2021). We retained genotype probabilities that had 100% confidence, allowing us to call genotypes and obtaining a VCF file with 21,726 single nucleotide polymorphisms (SNPs) suitable for analyses of the population structure and phylogenetic reconstruction.

### 2.4 Phylogenetic Reconstruction

We conducted three independent maximum likelihood analyses with IQ‐TREE (Nguyen et al. 2015) with the most appropriate model for our data selected using ModelFinder (Kalyaanamoorthy et al. 2017). As SNP‐based datasets only contain variable sites and can lead to branch length overestimation (Leaché et al. 2015), we applied ascertainment bias correction. We obtained support values for branches by running 100 bootstraps and applying a 25% burn‐in.

Concatenated approaches typically do poorly in the presence of ILS (Warnow 2015). An alternative approach, the multispecies coalescent model, offers a solution by inferring phylogenies while accounting for ancestral polymorphisms and gene tree‐species tree conflict (Edwards et al. 2016). We estimated species trees using two coalescent‐based methods: SNAPPER, implemented within BEAST2 (Bouckaert 2010); and SVDquartets, implemented within PAUP* (Swofford and Sullivan 2003) and is a method robust to the presence of gene flow (Long and Kubatko 2018). SVDquartets takes multi‐locus single‐site data, infers the quartet trees for all subsets of four species and then combines these trees into a species tree (Chou et al. 2015). We used SVDquartets to build 100,000 random quartet trees with the main genomic dataset (21,726 SNPs). We then explored uncertainty in relationships by producing 100 bootstrap replicates. We used Figtree (Rambaut 2009) to visualise a majority‐rule consensus tree, whereby only relationships that appear in at least 50% of rival trees are resolved. Since SNAPPER is more computationally intensive, we created 10 smaller datasets of 1500 SNPs each and ran independent analyses on each of them, combining the results with LogCombiner. We set two priors: (1) We time‐calibrated the root of the tree by applying a normal distribution with a mean at 2.25 Mya and an SD of 0.2, ensuring that 95% of the probability falls within the timeframe when *Zosterops* emerged (Leroy et al. 2021) and (2) we set a prior for the emergence of the Capricorn silvereye (*Z. l. chlorocephalus*) at 4000 years old with a standard deviation of 1000 as the islands that the subspecies currently inhabits were formed ~4,400 years ago and have been vegetated for no more than 4000 years (Clegg et al. 2008; Hopley et al. 2007). We ran Markov Chain Monte Carlo (MCMC) chains of 100,000 steps with a burn‐in of 10,000 steps. We visualised the full set of trees with DensiTree (Bouckaert 2010), highlighting inconsistencies from conflicting tree topologies.

To explore the occurrence of reticulate evolution we used Splitstree4 (Huson and Bryant 2006) and built a Neighbor‐Net, which visualises genetic distance between individual samples as a phylogenetic network (Bryant and Moulton 2004). This approach helps represent complex evolutionary histories that do not fit neatly into a bifurcating tree structure.

### 2.5 Population Structure

We performed a genomic principal components analysis (PCA) with PCAngsd and decomposed the resulting covariance matrix into eigenvectors to explore the population structure visually. We also conducted admixture analyses with NGSadmix (Skotte et al. 2013) by setting a maximum of 32 clusters, the total number of outgroup white‐eye species plus silvereye populations sampled. To explore substructure within two highly divergent silvereye groups, we created two separate datasets (Dataset 1: Southern Melanesia (SM) and Dataset 2: Australia, New Zealand and Outlying Islands [ANZO]) and repeated the PCA and admixture analyses described above, with 13 maximum clusters for each dataset. We used WGSassign (DeSaix et al. 2023) to assign individuals to populations. When populations are genetically distinct, individuals can be assigned to their population of origin with high confidence, whereas highly admixed populations result in low confidence assignments and many individuals are ‘misassigned’ to non‐origin populations. We defined silvereye populations at the level of island or continental region (each Australian state sampled, with samples from the Australian Capital Territory included with those from the state of New South Wales). We used a leave‐one‐out approach, removing one sample at a time from the sampled population, recalculating population's allele frequency and then estimating how likely it was for that sample to belong to each of the tested populations. Following DeSaix et al. (2024) we calculated posterior probabilities of assignment to a population by dividing the maximum likelihood of assignment over the sum of all likelihoods.

### 2.6 Gene Flow

In addition to ILS, admixture across highly divergent lineages could lead to phylogenetic conflict. We examined if admixture among continental subspecies explained unresolved nodes. We restricted this analysis to subspecies on the Australian continent and the large continental island of Tasmania because most widely used models assume constant population sizes and no selection (Smith and Hahn 2023), an assumption that is violated in small island silvereye populations that have experienced size fluctuations associated with colonisation and establishment (Estoup and Clegg 2003). We explored patterns of population splits and mixtures in the history of continental subspecies with Treemix (Pickrell and Pritchard 2012), which fits a model based on population allele frequencies and a Gaussian approximation to genetic drift. We generated maximum likelihood trees taking into account linkage disequilibrium by grouping 1000 SNPs into blocks. We tested a range of migration events (m from 0 to 15) and estimated the optimal migration edges with OptM (Fitak 2021), which resulted in a maximum of four migration events.

### 2.7 Morphological Distinctiveness

We trained a random forest classifier on a dataset of morphological data from 1292 individuals from 21 silvereye populations (Table S2) for which we had measured the following traits: wing, tail, metatarsal and head length, posterior bill length and anterior bill width and depth. The model training process was divided into three steps: (1) data resampling: to address the marked sample imbalance, we resampled the data with replacement so that each population had the same number of samples in the training set. This was done by grouping the data by population and sampling 15 instances from each group. This process was repeated 100 times to get a robust estimate of the variance in model performance on random subsets; (2) model training: we split the resampled data into training and testing sets, trained a random forest classifier on the training data and made predictions on the testing data. At each iteration, we added the confusion matrix of the predictions to an average confusion matrix and stored the feature importances to quantify uncertainty derived from stochasticity in both model performance and data selection; and (3) model evaluation: after training we evaluated the model's performance on the testing data. This included calculating accuracy, generating a confusion matrix and performing a permutation test. We generated several plots to visualise the model's performance, including a permutation test score plot (Figure S2), a confusion matrix heatmap, a hierarchical clustering of the confusion matrix and a feature importance plot (Figure S3).

The goal of this process was to train a model to classify populations based on their morphology, on the assumption that the degree of misclassification between pairs of populations is indicative of their overall morphological (dis)similarity based on a non‐linear combination of traits. This, in combination with the molecular data, can inform us of the extent to which genomic divergence is paralleled by morphological changes and highlight cases of either parallel/convergent morphological changes or morphological stasis.

### 2.8 Testing the Geographical Drivers of Population Differentiation

To explore whether water gaps among islands promote differentiation more than distances among continental sites, we built a Bayesian model in *brms* (Bürkner 2017) with pairwise F_ST_ as the response variable, representing population differentiation calculated with ANGSD, and predictor variables:Neighbor‐Net pairwise geographic distance between populations and whether the comparison was between island populations or between continental populations. We also tested for an interaction between the two predictor variables and added a multi‐membership grouping term to account for non‐independence in multiple comparisons. We used MCMC with four chains of 4000 iterations each, including a warm‐up of 400 iterations. We evaluated convergence via visual inspection of the MCMC trace plots, checking that ESS > 200 and that the *R* values for each parameter converged (*R* = 1 at convergence).

## 3 Results

### 3.1 Phylogenetic Analyses

We used several approaches to explore the evolutionary relationships: maximum‐likelihood with IQ‐TREE (Figure S4), coalescence model‐based SVDquartets with PAUP* (Figure 1B), and Bayesian inference with SNAPPER (Figure 1C). All analyses that included *Z. borbonicus* agreed on a split between this outgroup and South Pacific *Zosterops* species, estimated to have occurred around 2.22 Ma (95% credibility intervals (CI) = 1.83–2.61) by SNAPPER (Figure 1C; Figure S5). Of the other white‐eyes included, the Louisiade white‐eye *Z. griseotinctus* was the earliest group to branch off. According to IQ‐TREE and SVDquartets, *Z. flavifrons* and then *Z. tenuirostris* split after, followed by the silvereye clade, which contained the SM and the ANZO subclades. The SNAPPER tree suggested that all Southern Melanesian white‐eye species, including the silvereye, clustered together and split 1.5 Ma (95% CI = 1.11–1.92) from the ANZO cluster (those from Australia, New Zealand and the Outlying Islands, including Heron and Lord Howe Island), supported with a relatively lower posterior probability of 0.8 (Figure 1C, Figure S5). An alternative topology of silvereyes as a monophyletic group was also often observed in the SNAPPER tree (Figure 1C). The Neighbour‐Net suggested three main clades (Figure 3A): SM, ANZO, and other species of *Zosterops* but with Lord Howe Island more closely associated with other species of *Zosterops*.

The split between *Z. tenuirostris* and the three other non‐silvereye species in New Caledonia ( *Z. inornatus* , *Z. minutus* and *Z. xanthochroa* ) showed different patterns of relatedness depending on the analytical method used: SNAPPER supported that *Z. flavifrons* split first, followed by *Z. tenuirostris* (Figure 1C), while IQ‐TREE proposed placing it as its sister (Figure S4), and SVDquartets indicated a polytomy (Figure 1A). All analyses agreed that *Z. minutus* and *Z. xanthochroa* were more closely related than *Z. inornatus* and *Z. minutus* , which occur as single‐island endemics on Lifou. Shallow relationships in the phylogeny often showed lower support. For example, within Southern Melanesia, there were complex relationships: SVDquartets indicated a split between New Caledonia and Vanuatu populations (Figure 1A). However, SNAPPER suggested Tanna (Vanuatu) as the basal lineage, followed by Maré (New Caledonia) (Figure 1B), while IQ‐TREE suggested an early split of a clade containing Maré and Grand Terre (both New Caledonia) (Figure S4).

Another clade where low support was particularly noticeable was among mainland subspecies and recent colonisations in the ANZO group, with some relationships fully unresolved. Heron Island *Z. l. chlorocephalus* and Queensland *Z. l. cornwalli* individuals were variously identified as (i) part of the same clade (IQ‐TREE), (ii) a polytomy (SVDquartets, Figure S4), or (iii) having Heron Island as the sister to all mainland subspecies and recent island colonisations (SNAPPER) (Figure 1B). The relationships among mainland populations and recent colonisations varied depending on the method used, but some patterns emerged repeatedly: South and Western Australia were a single cluster, the North Island of New Zealand, Norfolk Island and French Polynesia were often grouped together, Chatham Island and the South Island of New Zealand were grouped together, and populations from Tasmania and the Eastern coast of Australia (Victoria and New South Wales) were part of the same clade.

### 3.2 Population Genomics

**FIGURE 2:**
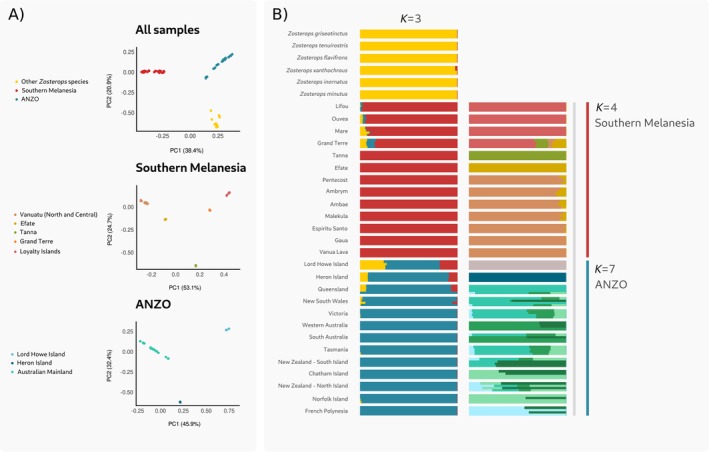
(A) Genomic PCA plots representing the two axes that explain most variation (PC1 on the *x*‐axis and PC2 on the *y*‐axis) for the three datasets all samples; southern Melanesian populations only; and a group consisting of Australian, New Zealand and outlying islands (ANZO). (B) NGSadmix bar plots for all samples (*K =* 3), Southern Melanesia (*K =* 4) and ANZO (*K* = 7). Each *K* is the most likely number of genetic groupings detected for each dataset. Within each population, each bar is the estimate of the individual's ancestry proportion from each of the assumed ancestral populations.

We visualised genetic population structure by plotting the two PCA axes that explained most genomic variation and the results from the admixture analysis. We repeated each analysis using three datasets: (i) the entire sample set including all silvereyes and white‐eye outgroups, (ii) a subset including silvereye samples from the ANZO group only and (iii) a subset including silvereye samples from Southern Melanesia only. Together, these analyses supported the existence of three major groups: Southern Melanesian silvereyes, ANZO silvereyes, and other *Zosterops* species. The first principal component in the PCA of the entire sample set explained 38.4% of the variation and separated Southern Melanesian silvereyes from all other silvereye populations and other *Zosterops* species, while the second (20.9%) separated all silvereyes from other *Zosterops* species (Figure 2A). Within Southern Melanesia, the silvereyes from the New Caledonian archipelago (Grand Terre and the Loyalty Islands—Ouvéa, Lifou and Maré) were distinct from those from North and Central Vanuatu islands, with Efate and Tanna populations intermediate between the two archipelagos (Figure 2A). These groupings, along with the distinctiveness of Tanna and Efate, were supported by admixture plots (Figure 2B). Assignment tests showed that individuals from Tanna, Efate, Gaua, Espiritu Santo and Pentecost were correctly assigned to their original populations (Table S3). In contrast, individuals sampled from other populations in the Central Vanuatu region were incorrectly assigned to Pentecost. Additionally, we observed a substantial level of population structure within the New Caledonian archipelago.

Within the ANZO group, the PCA revealed three distinct clusters of samples: Lord Howe Island, Heron Island and a larger cluster comprising samples from the Australian mainland, Tasmania and recently colonised populations (Figure 2A). This was supported by the admixture analysis, with the historically documented colonisations showing some finer substructure (Figure 2B) that was also evident in assignment tests (Table S3). The main patterns to note were Western Australia and South Australia samples clustered together, overlapping genetic profiles among widely separated eastern Australian sites (Queensland, New South Wales and Victoria), the continental island of Tasmania and high levels of admixture among recently colonised populations, except for Norfolk Island and French Polynesia that were more genetically distinct.

**FIGURE 3:**
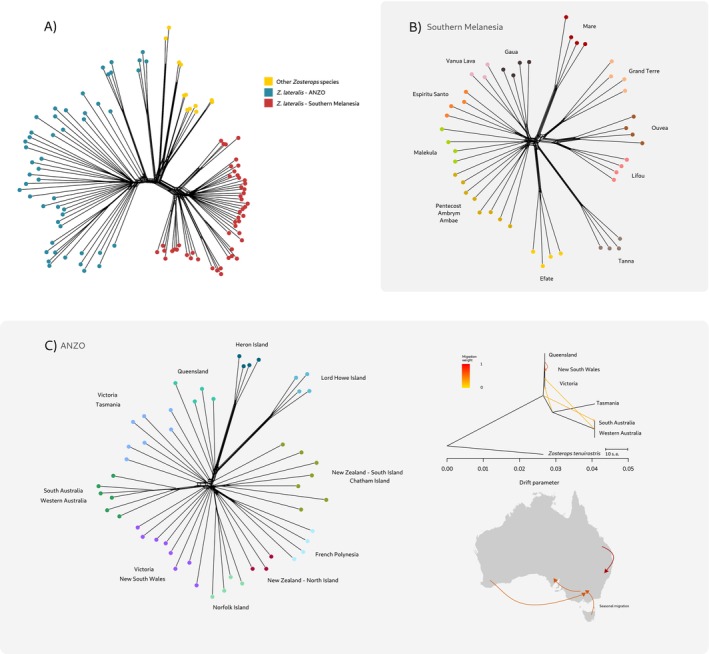
Phylogenetic network representing the evolutionary relationships and reticulate evolution across (A) silvereyes and other South Pacific *Zosterops* species, (B) Southern Melanesian silvereyes and (C) silvereyes from the ANZO cluster, which include continental species that show little clustering partly due to high levels of gene flow as shown in the TreeMix maximum‐likelihood tree on the right. The main directions of gene flow are shown on the map of Australia. Colours indicate different populations in panels A–C, while in the map (C), they represent migration weights.

Analysis of phylogenomic networks showed mild reticulation at the base of the ANZO subset, particularly among samples from Lord Howe Island, Heron Island and Queensland (Figure 3C). Reticulation was more evident in samples from South and Western Australia and Victoria and Tasmania, a pattern consistent with extensive gene flow across mainland populations, as supported by the Treemix results (Figure 3C). We found a star‐like pattern where many edges radiate outward from a single central node across the mainland and recent colonisations, which often indicate a recent radiation or extensive gene flow. In Southern Melanesia, islands in New Caledonia showed clear independent lineages divided by island but with reticulation between Ouvéa and Lifou and Maré and Grand Terre (Figure 3B). Similarly, Tanna and Efate showed some reticulation but were primarily independent lineages. In central Vanuatu, only Espiritu Santo showed some lineage independence, as did Gaua and Vanua Lava populations in northern Vanuatu.

### 3.3 Morphological Differentiation

Training a random forest classifier on morphological data revealed that some island populations, such as those from Lord Howe Island, Tanna and Grand Terre, could be accurately distinguished from other silvereye populations based on their morphometrics alone (Figure S3; Table S4). Other Southern Melanesian populations sometimes clustered together, especially across Central Vanuatu (Malekula, Espiritu Santo, and Pentecost) and two of the Loyalty Islands (Lifou and Ouvéa). The Heron Island population clustered with islands from Vanuatu despite not being close genetically, indicating convergence. In line with the genetic results, it was harder to morphologically distinguish among recently colonised island populations and among those sampled on the Australian mainland.

### 3.4 Water Barriers but Not Continental Distances Promote Genetic Population Differentiation

**FIGURE 4:**
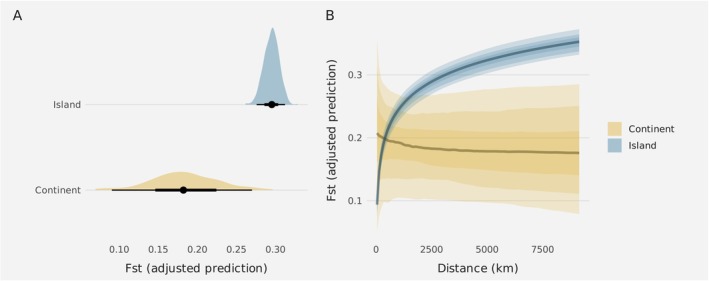
(A) Adjusted predictions estimated with *brms* of *F*_ST_ on islands and the continent. (B) Adjusted predictions of the effect of the distance on *F*_ST_ levels among continental populations (yellow) and islands (blue). The uncertainty bands are darker for lower values and lighter for higher values, representing 50%, 80% and 95% credibility intervals.

F_ST_ was greater between island populations than between continental populations (Figure 4A; marginal effect at the mean = −0.88; 95% CI [−1.8, −0.034]; Posterior probability = 0.95). *F*_ST_ increased with distance on islands but not on the continent, although the latter showed wide credibility intervals (Figure 4B).

## 4 Discussion

Whole genome sequencing illustrates how divergence patterns are heavily influenced by the geographic settings in the silvereye. In continental silvereyes, large geographic distances and biogeographic barriers do not result in a strong population structure and high population connectivity is evident from star‐like and reticulated patterns among geographically widely separated populations and subspecies. Hence, subspecies from the continent are often highly admixed, with relatively low levels of morphological distinctiveness. However, in island settings, the same species was characterised by pronounced structure; geographically close island populations were often highly distinct, demonstrating that even short water gaps can be significant barriers. Parallel to the much stronger island population genetic structure, morphological distinctiveness was often high. Together, these differences in genomic and phenotypic patterns suggest lower population connectivity in island‐distributed versus continentally distributed silvereyes, and therefore, the generation of the great speciator pattern emerges from a reduction in dispersal in island environments.

### 4.1 The Generation of a Great Speciator Pattern in Silvereyes

Great speciators present a paradox: they are highly dispersive—able to colonise multiple islands—yet show strong genetic and phenotypic divergence among populations, which typically requires limited gene flow. The proposed resolution invoked a reduction in dispersal capacity after island colonisation, driven by strong selection against dispersal when over‐water dispersal becomes a risky strategy (Diamond et al. 1976). The silvereye case supports this premise. Island populations show more pronounced population genetic and phenotypic structure than continental ones, and observations of silvereye movements support the idea that these patterns result from a shift in dispersal rather than a pre‐existing reluctance to cross water gaps for the species. For example, a portion of the Tasmanian silvereye population crosses the 240 km stretch of Bass Strait to and from the Australian mainland each winter (Chan and Kikkawa 1997); continental silvereyes are often observed on Heron Island, 80 km from the coast (Kikkawa 1970; Sendell‐Price et al. 2020), and multiple long‐distance overwater colonisation events (800 to 2000 km) have been recorded in the last 200 years (Clegg, Degnan, Kikkawa, et al. 2002). A shift in dispersal is also consistent with observations that older and more sedentary island silvereye populations have experienced changes at genes associated with dispersal characteristics when compared with continental and recently colonised populations (Estandía, Sendell‐Price, Oatley, et al. 2023). Because most models of divergence and speciation require a reduction in gene flow (Coyne and Allen Orr 1998), reduced dispersal provides the conditions under which microevolutionary processes of drift and selection can then be more effective in producing divergence. Local adaptation could also be a barrier to successful dispersal and establishment, so‐called isolation by adaptation (Spurgin et al. 2014). Alternatively, isolation by colonisation (Spurgin et al. 2014), where founder effects contribute to a stronger population genetic structure in silvereyes on islands versus continents, could occur if each island population was established from genetically non‐representative flocks with insufficient ongoing gene flow among populations to homogenise the genetic structure. This would have the effect of producing essentially instantaneous population structure. However, studies of the degree of genetic differentiation attributed to population founding suggest that this is unlikely in most cases because of often substantial founding flock sizes and rapid recovery from small populations (Estoup and Clegg 2003; Sendell‐Price et al. 2021).

The generation of a great speciator pattern is likely to be modulated by factors other than reduction in dispersal per se, including the timing of island formation and their spatial arrangement, along with patterns of population persistence and recolonisation if local extinctions occur (Estandía 2023). Wherever the timing of island formation coincides with the spread and divergence of a species, there will be more opportunities to take early advantage of niche availability (Grant and Grant 2008; Lack 1947) and to do so before the onset of dispersal reductions. While some islands in southern Melanesia long pre‐dated the silvereye's arrival (e.g., Grand Terre, Espiritu Santo and Tanna), the species expansion through the region coincided with a time of increased island formation and growth in the last one to two million years (Maurizot et al. 2020; Monjaret et al. 1991). The spatial arrangement of islands influences source‐sink dynamics and population connectivity patterns. In silvereyes, there are populations at the periphery of archipelagos (e.g., Southern Vanuatu and the Loyalty Islands), where genetic and morphometric diagnosability are high. More centrally located islands in the Vanuatu archipelago display weaker population structure and evidence of reticulate evolution, suggesting that they are well‐connected by gene flow, confirming inferences from microsatellite DNA (Clegg and Phillimore 2010). Hence, the spatial arrangement of islands within southern Melanesia, along with the occurrence of silvereyes on some highly isolated islands such as Lord Howe Island, provides multiple opportunities for peripheral population divergence. Lack of population persistence can limit avian diversification (Diamond and Marshall 1977; Ricklefs and Bermingham 2007) and therefore inhibit the formation of a great speciator pattern. Numerous silvereye populations have clearly persisted for long time periods. While exact dates should be treated with caution, the Southern Melanesian silvereye populations as a group are old, in the order of 1.3 Mya, and other peripheral populations such as Lord Howe Island, in the order of 0.8 Mya. Their population resilience can be partly attributed to the ability to rapidly recover from small population sizes, for example, evidenced after cyclones on Heron Island (Kikkawa 2003) and following the historically recorded colonisation of Norfolk Island (Mees 1969). Wherever localised island extinctions occur, they are likely to be eventually replaced by a new colonisation to restart the process of divergence. Hence, along with a capacity to shift to lower dispersal in island situations, the silvereye has several other life history characteristics that are conducive to a great speciator pattern arising.

Whether the silvereye stays in a great speciator stage or progresses to subsequent stages of a taxon cycle, forming clearly defined, island‐restricted species, cannot be known with certainty. However, it could be argued that some island silvereye subspecies already hover at the species boundary and are sometimes referred to as full species for example *Z. l. chlorocephalus* (Kikkawa 2003) and *Z. l. tephropleurus* (see Mees 1969; McArthy 2006). To better understand the generalities of formation and stability of the great speciator stage, we should combine modelling approaches that incorporate a range of parameters (e.g., species traits relating to dispersal and spatial arrangement of islands and archipelagos) that produce the pattern (Estandía 2023), comparisons of drivers of great speciator patterns across multiple species and collection of more empirical data on the morphological, behavioural and genetic signatures of changes in dispersal (Estandía, Sendell‐Price, Oatley, et al. 2023).

While multiple sources of information from the silvereye system indicate that reduced dispersal and gene flow in the island context is key to the generation of a great speciator pattern, an alternative explanation of intermediate dispersal could be considered for other great speciators, especially where a continental comparison is not available. The few studies that have examined genetic and phenotypic divergence among island populations compared to continental ones in the same species show consistently higher divergence among island populations, for example, Pacific and Welcome Swallows (Broyles et al. 2023). While these examples are not (or not necessarily) cases where a great speciator stage has emerged, they serve to show that it may indeed be reasonable to assume a change in dispersal propensity in the island context.

### 4.2 Silvereye Origins and Relationships Among Silvereye Subspecies and Populations

Our phylogenomic and population genomic analyses provide the best resolution of evolutionary relationships among silvereye populations to date. The silvereye likely diverged from other South Pacific white‐eyes approximately 1.5 Mya. The Zosteropidae radiation occurred within the last 2 Mya, initiated in Asia (Gwee et al. 2020), and the speed of this radiation means that much was occurring in parallel; for example, the silvereye divergence time is comparable to that between the Afrotropical and Australasian *Zosterops* and to that of silvereye from other *Zosterops* species (Martins et al. 2020; Vinciguerra et al. 2023; Wickramasinghe et al. 2017).

Our analyses resolve evolutionary relationships among silvereyes and allow us to assess the validity of the silvereye as a single species and the separation into morphological subspecies—an important consideration for recognising great speciators. The first consideration is whether Southern Melanesian silvereyes can be considered a separate species from those in Australia, New Zealand, and outlying islands. They are phylogenetically and phenotypically distinct, but whether this is sufficient for species status would require further information on species isolation mechanisms, such as bird song or plumage. While some subspecies show clear morphological differences, overall diagnosability is limited, even across island populations. This likely reflects the interplay between selection—particularly predictable shifts like those seen under the island rule (Estandía, Sendell‐Price, Oatley, et al. 2023; Estandía, Sendell‐Price, Robertson, et al. 2023)—and other evolutionary forces such as gene flow and drift. In island populations, reduced dispersal facilitates divergence, but the extent of morphological differentiation ultimately depends on how selection interacts with connectivity, colonisation history, and population size.

Silvereyes of Southern Melanesia (New Caledonia and Vanuatu) show biogeographic patterns that do not fully align with the identity of morphological subspecies. An example is seen in the New Caledonia Loyalty Islands, where *Z. l. nigrescens* has a disjunct distribution on Maré and Ouvéa, interrupted by *Z. l. melanops* on Lifou. However, silvereyes on Maré and Ouvéa cannot be considered the same subspecies based on phylogenomic relationships that instead show that Maré (and Grand Terre) silvereyes are basal to those on Lifou and Ouvéa, with extensive reticulation likely indicating gene flow between the latter pair of islands. Hence, the diversification pattern of Loyalty Island silvereyes follows the timing of increasing emergent land availability starting approximately 2 Mya, when pre‐existing Pliocene paleo‐atolls were uplifted, starting with Maré, and followed by Lifou then Ouvéa (Maurizot et al. 2020). Geographic patterns of genomic variation across the Vanuatu archipelago also deviated from expectations of morphological subspecies. The geographic border between the northern subspecies, *Z. l. tropica* (designated from Espiritu Santo northward), and the southern subspecies, *Z. l. vatensis* (from Malekula southward) (Mees 1969) did not align to the genomic groupings that instead showed only southern islands of Efate and Tanna to be distinct from a northern group and from each other. This genomic pattern agreed with groupings identified by microsatellite variation (Clegg and Phillimore 2010). In addition to its genomic distinctiveness, the Tanna population was highly morphologically distinct, supporting its separate subspecies status as *Z. l. macmillani* (Clements et al. 2021; Mayr 1937). Two isolated silvereye subspecies that display high morphological and genomic distinctiveness are *Z. l. tephropleurus* on Lord Howe Island and *Z. l. chlorocephalus* on the Capricorn Bunker Islands of the southern Great Barrier Reef. As mentioned previously, some authors have treated these as full species (Kikkawa 2003; Mees 1969); however, our analyses place both well within the currently recognised silvereye grouping, and raising either to a species status would result in paraphyly unless Southern Melanesian silvereyes were also considered different species.

Continental and Tasmanian silvereyes display high genetic connectivity among populations and across morphological subspecies designations. Connectivity patterns were often consistent with known movement patterns and band returns across vast distances. For example, east coast silvereyes were highly admixed to the point where individuals could not be confidently assigned to their original locations. The three subspecies that occur from Tasmania (*Z. l. lateralis*), Victoria (*Z. l. westernensis*) and New South Wales and Queensland (*Z. l. cornwalli*) interact during the partial winter migration of the two southerly subspecies (Chan 1995), and gene flow likely results as individuals fail to return south for breeding. Silvereyes from Western Australia (*Z. l. chloronotus*) and South Australia (*Z. l. pinarochrous*) are separated from each other and the eastern Australian subspecies by major biogeographical barriers. These barriers are the Nullarbor Plain, a desert that stretches approximately 1200 km between Western and South Australia, and the Eyrean barrier, a historical biogeographical barrier in South Australia (Ford 1987). *Z. l. chloronotus* and *Z. l. pinarochrous* sampled over 2000 km apart and separated by the Nullarbor Plain were genetically similar, while *Z. l. pinarochrous* and *Z. l. westernensis* sampled over 1000 km apart and separated by the Eyrean barrier while still admixed to some extent showed greater differentiation. These patterns are consistent with studies that suggest the Nullarbor Plain has had a lower impact on current avian diversity patterns compared to the Eyrean barrier (Dolman and Joseph 2015).

We did not recover a strong phylogenetic signal mapping to the historically recorded natural colonisation sequence of *Z. l. lateralis* from Tasmania to New Zealand (South Island (1830s), North Island and Chatham Island (1856)) and Norfolk Island (1904) (Mees 1969), and the human‐mediated introduction from South Island to Tahiti (French Polynesia) (1937) (Guild 1938). Instead, the genetic relationships among the continental, Tasmanian, and recently colonised island silvereye populations mentioned above show the expected phylogenetic signal of paraphyly following peripatric divergence of small populations derived from a widespread parent group (Freudenstein et al. 2017; Wootton et al. 2023). Rapid morphological evolution in novel environments is known across vertebrate taxa (Millien 2006; Nicholson et al. 2023; Valentin et al. 2018) and recently colonised silvereye have diverged morphologically (Clegg, Degnan, Kikkawa, et al. 2002; Estandía, Sendell‐Price, Robertson, et al. 2023; Sendell‐Price et al. 2020) but we showed here that these differences are not sufficient to be diagnostic of populations.

While the phylogenomic treatment of silvereyes has shed light on many aspects of their evolutionary history, numerous uncertainties remain because of the rapid diversification, limitations of the methods used, and potential for hybridisation to complicate relationships. For instance, the exact timing of splits from the DensiTree should be treated with caution because a strict molecular clock, as imposed by SNAPPER, assumes a constant rate of evolution across all lineages. However, this assumption may not be appropriate for island populations, where factors like founder effects, selective pressures, and gene flow can cause rate heterogeneity across populations, making the resulting divergence times potentially less robust. Some dates are inconsistent with previous information, for example, the divergence time for Heron Island *Z. l. chlorocephalus* was estimated at 80 thousand years, whereas recent divergence times indicated by mitochondrial data (Degnan and Moritz 1992) and geological data estimating that the island has been vegetated for 4000 years (Hopley 1982) have been used to cap the age of the subspecies (e.g., Clegg et al. 2008). The phylogenies produced also raised questions whether continental silvereyes were the ancestral source for all island populations, as has been suggested previously (Black 2010; Mees 1969). Our time‐calibrated phylogeny suggests that some of the oldest lineages are from Pacific islands, raising the possibility of island to continent colonisation as described in other organisms, such as in other species of birds (Filardi and Moyle 2005; Lapiedra et al. 2021) and in *Anolis* lizards (Patton et al. 2021). In the south‐west Pacific, colonisation is expected to mainly occur eastward from the larger landmasses of Australian and New Guinea, but there are also exceptions, for example, ‘upstream’ colonisation by corvoid birds (Jønsson et al. 2011). The complicated colonisation history of the silvereye requires further examination, potentially using mitogenome‐based phylogenies where events can be more readily dated. Finally, hybridisation following secondary contact with congeneric white‐eyes, or transient gene flow between highly divergent subspecies, may explain some of the reticulation patterns observed, including the conflict in the DensiTree topology, where Southern Melanesian silvereyes either form a monophyletic group or cluster more closely with other *Zosterops* species from the same region potentially due to long‐term coexistence and introgression with other *Zosterops* species. Another factor that could contribute to the low nodal support observed in some clusters is the possible extinction of key lineages (Quental and Marshall 2010). Apart from ongoing or historical gene flow that can account for weak phylogenetic resolution, it is also plausible that the absence of certain lineages—now extinct and therefore missing from the analysis—could obscure true evolutionary relationships. The incompleteness of the fossil record could lead to misleading signals in the tree topology and reduce support at deeper nodes, especially in cases where extinct lineages may have played a role in diversification. Such possibilities complicate the retrieval of unequivocal evolutionary relationships.

### 4.3 Comments on Relationships Among White‐Eye Outgroups

Taxonomically well‐sampled white‐eye phylogenies have been produced elsewhere (Oliveros et al. 2021; Vinciguerra et al. 2023); however, one of our outgroups, *Z. minutus* , from Lifou, New Caledonia, has not previously been included in these published analyses. Our analyses place this species as sister to *Z. xanthochrous* from Grand Terre, New Caledonia. The relatively close relationship between single island endemics of *Z. inornatus* from Lifou and *Z. tenuirostris* from Norfolk Island was consistent with a mtDNA analysis (Vinciguerra et al. 2023). However, in our analyses, whether *Z. tenuirostris* was basal (DensiTree) or sister to (IQ‐TREE) *Z. inornatus* was uncertain. Some *Zosterops* species are known to hybridise (Gwee et al. 2020; Manthey et al. 2020) and the possibility of hybridisation within sympatric Norfolk Island *Zosterops* species (Gill 1970) could introduce uncertainty around the exact relationship. Our genome‐wide data also confirmed that sympatric insular *Zosterops* species on islands in New Caledonia and Vanuatu and on Norfolk Island are not closely related. High levels of dispersal by members of the family likely resulted in many tests of sympatry, not all of which would have been successful. In addition to their relatively distant genetic relationships, existing cases of sympatry in our sample can show striking body size differences. For instance, *Z. inornatus* is among the largest of white‐eyes (mean ± SD: 21.6 ± 1.27 g, *N* = 41) and *Z. minutus* among the smallest (9.0 ± 0.9 g, *N* = 186), and these single island endemic species on Lifou live in sympatry with the middle‐sized, single island endemic silvereye subspecies *Z. l. melanops* (14.06 ± 1.32 g, *N* = 82); in comparison, the silvereye on a neighbouring island of Tanna ~270 km to the north‐east of Lifou approaches the size of *Z. inornatus* (19.4 ± 2.22 g, *N* = 12) (SMC unpublished data). Evolution towards larger body size is frequently observed in insular *Z. lateralis* (Clegg, Degnan, Moritz, et al. 2002; Clegg et al. 2008; Estandía, Sendell‐Price, Robertson, et al. 2023); however, the extent of body size increase may be constrained by the presence of particularly large‐bodied white‐eyes in some cases.

## 5 Conclusions

Our study of silvereye phylogenomics and population genomics reveals differences between continental and island populations, with mainland forms showing a low genetic structure and high connectivity despite large geographic distances, while island populations exhibit strong genetic and morphological divergence even over short distances. This pattern provides evidence that water barriers are more effective than continental distances at restricting gene flow, supporting the hypothesis that great speciator patterns can arise from a reduction in dispersal propensity following island colonisation. Despite using whole‐genome data and multiple analytical approaches, some phylogenomic relationships remain uncertain, highlighting challenges in reconstructing evolutionary histories in rapidly radiating groups. The silvereye system demonstrates how geographic context fundamentally shapes divergence, with the interplay between dispersal ability and isolation determining when and where speciation processes advance. These findings contribute to our broader understanding of how biodiversity is generated across fragmented landscapes and island archipelagos worldwide.

## Author Contributions

Conceptualization: A.E., S.C., and B.C.R.; data curation: A.E.; formal analysis: A.E. (bioinformatics), N.M.R. (morphology); funding acquisition: S.C. and B.C.R.; investigation: A.E.; methodology: A.E., N.M.R.; project administration: A.E., S.C.; resources: A.E., A.T.S.‐P., D.A.P., W.G., B.C.R., and S.C.; supervision: S.C.; visualisation: A.E.; writing – original draft: A.E. and S.C.; writing – review and editing: A.E., N.M.R., S.C., D.A.P., A.T.S.‐P., and B.C.R.

## Conflicts of Interest

The authors declare no conflicts of interest.

## Supporting information


Figure S1

Table S1

## Data Availability

Code to reproduce all analyses is available at https://github.com/andreaestandia/phylogeny_silvereye and https://github.com/nilomr/silvereye‐morphology. Raw sequencing data are available at the NCBI Sequence Read Archive under BioProject PRJNA1264480, and VCF and BEAGLE files have been deposited in Dryad: https://doi.org/10.5061/dryad.vdncjsz69.
