# Supplementary material for: Islands Promote Diversification of the Silvereye Species Complex: A Phylogenomic Analysis of a Great Speciator
Source: Mol Ecol. 2025 Jun 11;34(14):e17830. doi: 10.1111/mec.17830 (PMC12237087; doi:10.1111/mec.17830)
Supplement: Supplementary file 1 — Figure S1 [file MEC-34-e17830-s002.pdf]

MOLECULAR ECOLOGY

Supplemental Information for:

Islands promote diversification of the silvereye species complex: a phylogenomic analysis of a great speciator

Andrea Estandia[1], Nilo Merino Recalde[1], Ashley T. Sendell-Price[1], Dominique A. Potvin[2], William Goulding[3,4] , Bruce C. Robertson[5], Sonya Clegg[1,3]

[1]Edward Grey Institute of Field Ornithology, Department of Biology, University of Oxford, Oxford, United Kingdom
[2]School of Science, Technology and Engineering, University of the Sunshine Coast, Petrie, Australia
[3]School of Environment and Science, Griffith University, Nathan, Australia
[4]Biodiversity Program, Queensland Museum Kurilpa, Brisbane, Australia
[5]Department of Zoology, Otago University, Dunedin, 9054, New Zealand

MOLECULAR ECOLOGY

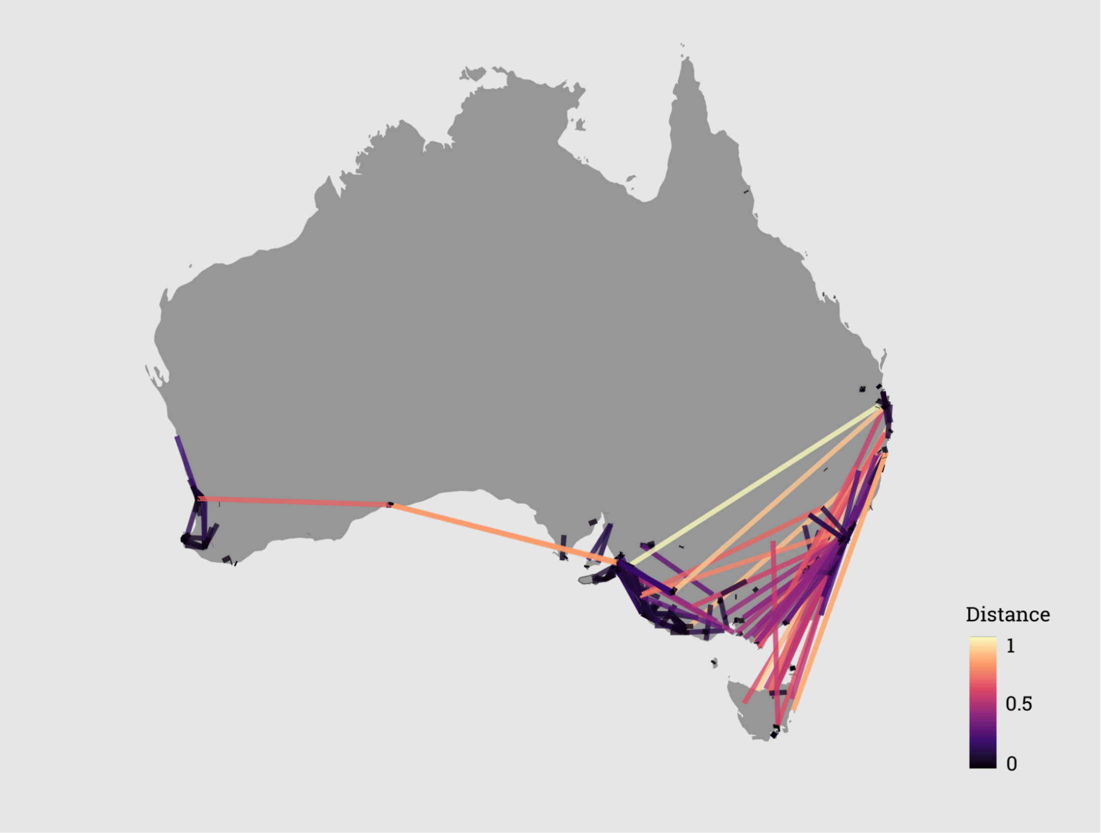

Figure S1. Silvereye movement patterns based on banding recovery records between 1956 and 2015 provided by the Australian Bird and Bat Banding Authority. Lines are coloured by distance (normalised) between where the bird was first ringed and where it was caught again. Note that population density and banding effort vary greatly across space and time.

MOLECULAR ECOLOGY

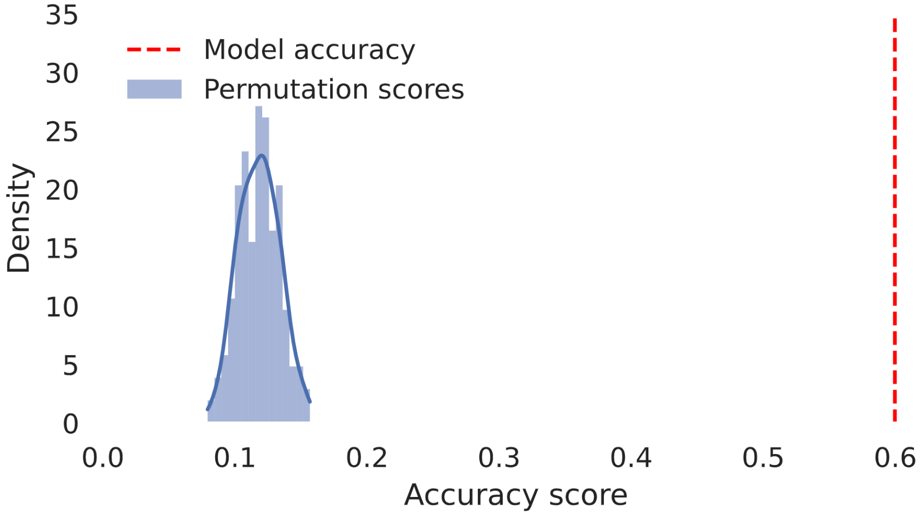

Figure S2. Accuracy of morphological classification to correct sampled population under a random permutation model (blue probability density distribution) compared to our average classification accuracy (red dashed line; ~60%).

MOLECULAR ECOLOGY

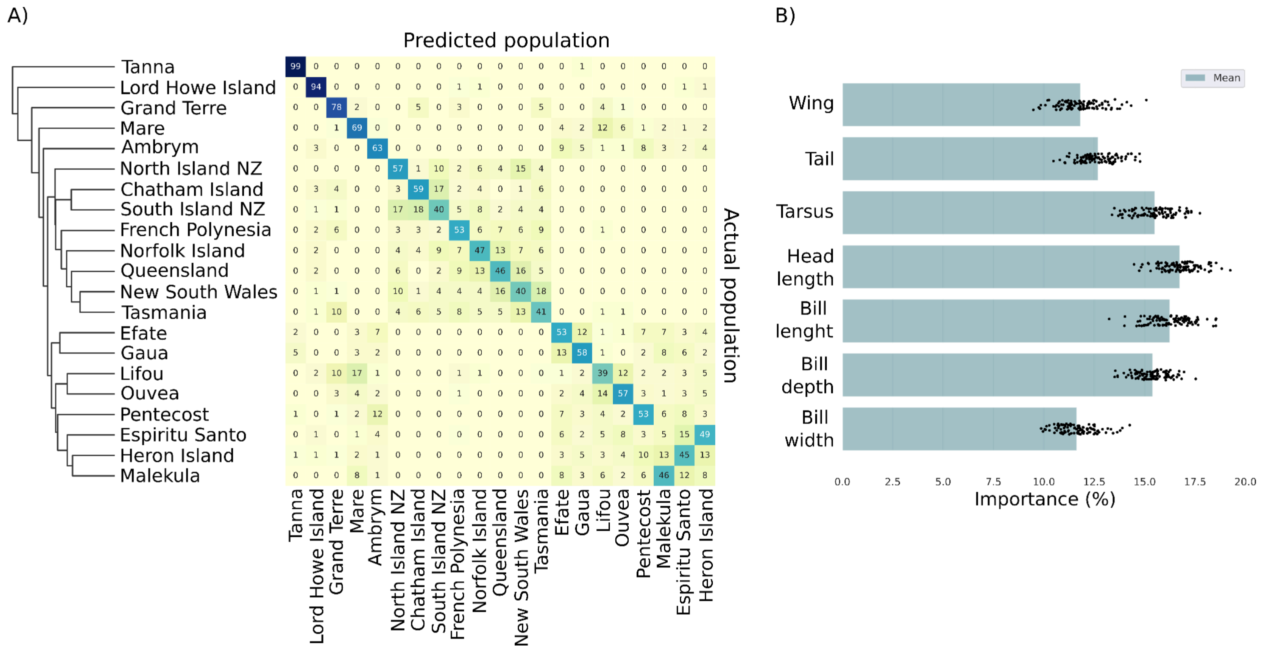

Figure S3. A) Morphological distinctiveness of populations determined by hierarchical clustering of the Random Forest-based confusion matrix. The diagonal shows the percentage of iterations the predicted population matched the sampled population, and the off-diagonal values cases of mis-assignment. Cells are shaded from yellow (low percentage) to dark blue (high percentage). The Tanna population in Vanuatu shows high morphological distinctiveness with 99% of the island sample assigned to itself, whereas correct assignment occurred around 40% of the time in a suite of other populations e.g. New South Wales mis-assignments to distant locations of Tasmania (18%) and Queensland (16%), and the island of Lifou with mis-assignments to close neighboring islands of Maré (17%) and Ouvéa (12%); B) All traits are similarly important in determining the morphology.

MOLECULAR ECOLOGY

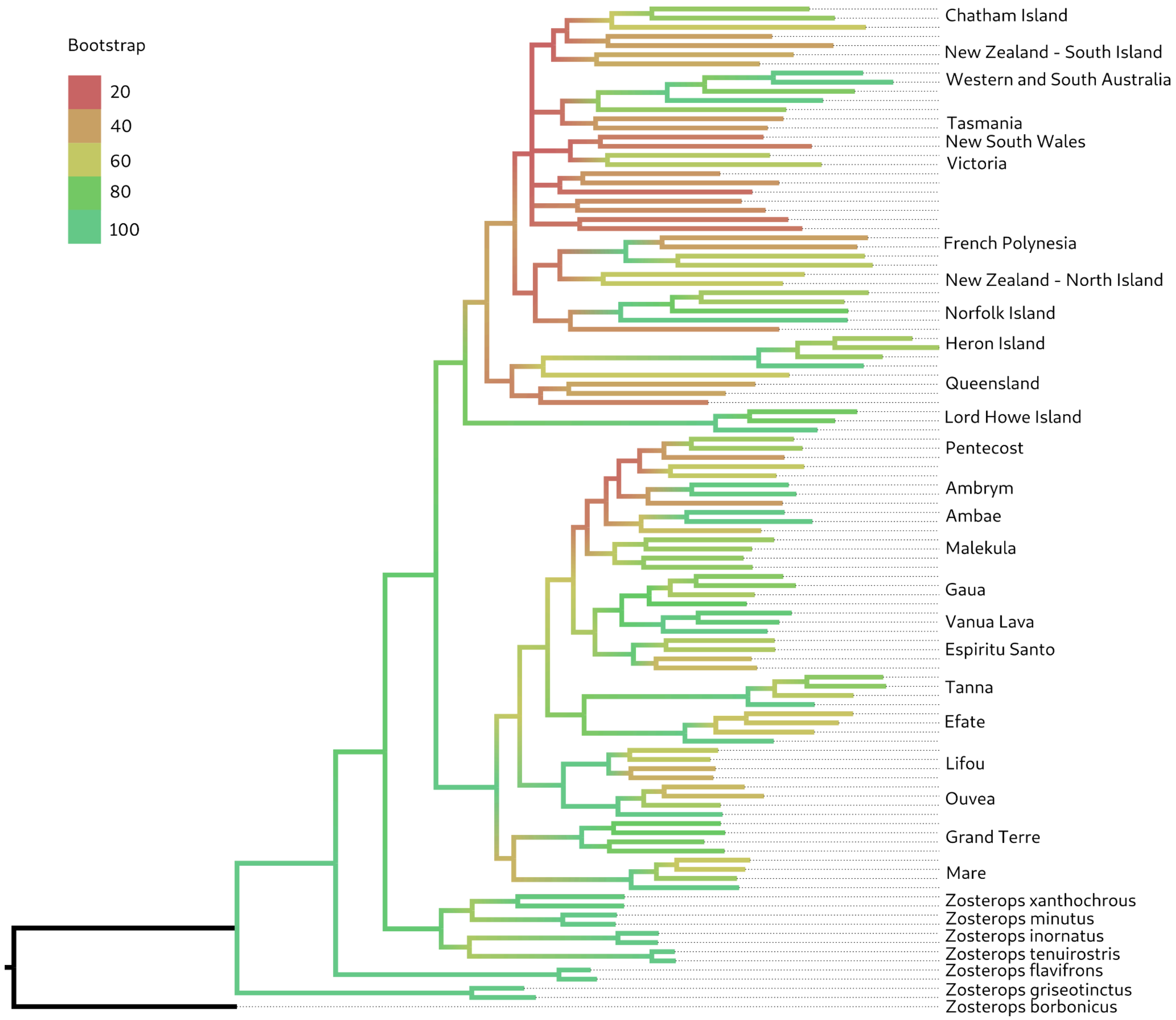

Figure S4. The phylogenetic tree generated from IQTREE, where each branch is colour-coded to indicate the bootstrap support for its parent node. This reveals low bootstrap support and therefore significant uncertainty across the ANZO cluster (excluding Lord Howe Island) and some islands of north and central Vanuatu in the Southern Melanesia cluster.

MOLECULAR ECOLOGY

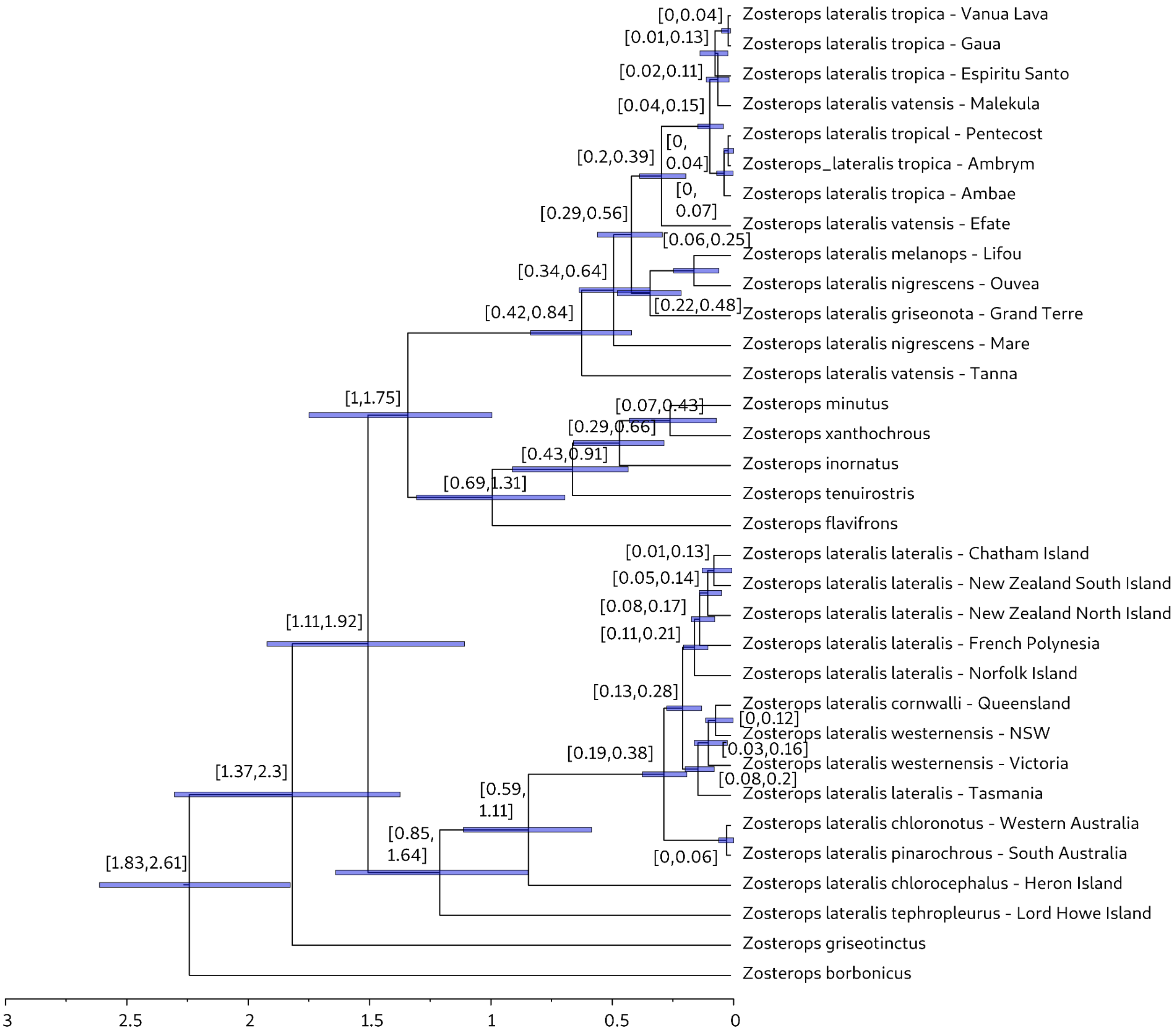

Figure S5. Maximum clade credibility representing the consensus topology and the 95% credibility intervals for the age estimate in Mya generated with SNAPPER.

MOLECULAR ECOLOGY

Supplementary tables captions

Table S1. Curated dataset of the samples sequenced and associated metadata.

Table S2. Curated dataset of the samples used for the morphological analysis.

Table S3. WGSassign results.

Table S4. Mean and SD of the morphological traits by population.
